# A Naturally Occurring Feline Model of Head and Neck Squamous Cell Carcinoma

**DOI:** 10.1155/2013/502197

**Published:** 2013-07-16

**Authors:** Jackie M. Wypij

**Affiliations:** Department of Veterinary Clinical Medicine, University of Illinois at Urbana-Champaign, Hazelwood Drive, Urbana, IL 61802, USA

## Abstract

Despite advances in understanding cancer at the molecular level, timely and effective translation to clinical application of novel therapeutics in human cancer patients is lacking. Cancer drug failure is often a result of toxicity or inefficacy not predicted by preclinical models, emphasizing the need for alternative animal tumor models with improved biologic relevancy. Companion animals (dogs and cats) provide an opportunity to capitalize on an underutilized and biologically relevant translational research model which allows spontaneous disease modeling of human cancer. Head and neck squamous cell carcinoma (HNSCC) is a common cancer with a poor prognosis and limited clinical advancements in recent years. One potential novel spontaneous animal tumor model is feline oral squamous cell carcinoma (FOSCC). FOSCC and HNSCC share similar etiopathogenesis (tobacco and papillomavirus exposure) and molecular markers (EGFR, VEGF, and p53). Both human and feline SCCs share similar tumor biology, clinical outcome, treatment, and prognosis. Future clinical trials utilizing FOSCC as a tumor model may facilitate translation of preclinical cancer research for human cancer patients.

## 1. Introduction

Advances in our understanding of cancer at the molecular level have outpaced clinical application of this information to human patients. It can take more than a decade and $800 million dollars to develop a new drug or diagnostic agent, yet less than 10% of promising drugs achieve FDA-approval [1, 2]. This discouraging drug failure rate is in part due to limited predictive value for drug toxicity and efficacy of accepted preclinical models [2]. The value of pre-clinical models to subsequently predict drug success is inherently based on the relevancy of animal models. Murine models provide crucial opportunities to investigate specific molecular and genetic pathways. These models often incorporate chemically induced cancer or xenografted human cancer cell lines in immunosuppressed animals. Thus, despite their importance these conventional murine models fail to adequately characterize the biologic variations inherent in spontaneously arising human cancer such as long latency period, genomic instability, tumor heterogeneity, and the complexity of the tumor microenvironment. While no single model can provide solutions to all drug-development questions, integration of information from multiple modeling systems can improve the successful translation of a novel drug into human patients. One approach is capitalization on biologically relevant companion animal translational research which allows spontaneous disease modeling with similarities to human cancer that cannot be recreated in induced models [3–6].

*Benefits and Limitations of Companion Animal and Murine Models for Cancer Research [3, 4, 4, 5, 5–9]*

*(1) Cancer-Bearing Pets*

- Positive Attributes/Similarities to Human HNSCC
  - high incidence in native population
  - access to normal and cancer-bearing animals
  - immune competence
  - spontaneous tumor development
  - shared environment/diet/risk exposure
  - similar tumor biology and genetics
  - similar age, sex
  - shared molecular targets
  - proof-of-target analysis
  - recapitulates tumor microenvironment and cell-stromal interactions
  - tumor heterogeneity
  - similar latency period
  - acquired resistance
  - spontaneous recurrence and metastasis
  - similar prognostic factors
  - similar diagnostics and cancer therapy
  - multimodality treatment feasible
  - serial biopsies/biospecimens feasible
  - increasing species-specific reagents → gene-expression analysis and tumor proteomics
  - ability to validate biomarkers
  - not constrained by traditional Phase I/II/III trial design
  - pet trials similar in caliber yet less costly than human trials
  - minimally pretreated with good performance scores
  - good owner/client compliance

*(2) Murine Limitations*

- Positive Attributes
  - conventional model, well accepted
  - ability to manipulate gene expression
  - crucial in evaluating molecular paths
-  Limitations
  - induced disease
  - tumor homogeneity
  - inbred population
  - artificially shortened lifespan
  - controlled, protected environment.

Traditionally, promising *in vitro* compounds are tested in human-xenografted immunocompromised mice to obtain efficacy and toxicity data in least sentient species. Putative anticancer drugs demonstrating preclinical effectiveness are then tested in primates to assess toxicology, followed by clinical testing in humans. Ethical concerns and recent funding restrictions on nonhuman primate research intensify the need to find alternative animal tumor models. Companion animal models (pets dogs or cats) could provide an essential intermediary safety and efficacy step between artificially induced murine tumor models and human patients. Sixty percent of US households own a pet, with 64% owning ≥2 pets, thus placing approximately 75 million dogs and 85 million cats at risk for cancer [10]. Estimates of age-adjusted overall cancer incidence rates per 100,000 individuals/year at risk are 381 for dogs and 264 for cats [3]. A large number of pets are affected with cancer each year, representing an underutilized resource in translational oncology research. Pet animals have been used in other fields of biomedical research based on similarities in physiology, anatomy, and drug metabolism. Cats in particular have been well studied as models of HIV and viral infections, diabetes mellitus, and obesity [11–14]. 

Pet owners are motivated to seek cancer treatments to both prolong their pets' lives as well as maintain the quality of life. Pet owners frequently seek out specialized care from board-certified veterinary oncologists, both in private practice and at teaching institutions. Multimodality treatments are consistently available, including surgical oncology, chemotherapy, immunotherapy, and radiation therapy. However, effective “standards of care” do not exist for many types of cancer in companion animals, providing opportunities to implement experimental therapies. Companion animal owners are interesting in pursuing clinical trials to evaluate novel diagnostic and therapeutic modalities, motivated by several potential benefits: for their individual pet, for other pets and pet owners faced with a similar challenge, and translation to benefit human cancer patients. Clinical trials customarily provide financial incentives, which are appealing given the high out-of-pocket expense for conventional treatments given the paucity of health insurance coverage in veterinary medicine. As most clinical trials are designed and administered by veterinary oncologists, they focus on clinical medical applicability and are not terminal studies. All institutional clinical trials are performed under both the auspices of the Institutional Animal Care and Use Committee (IACUC) and the participating veterinary hospital's standard operating procedures. Informed client consent and voluntary participation on part of the pet owner are key aspects. Many veterinary cancers are identified at an advanced stage with limited conventional treatment options, and thus experimental therapies are very acceptable to pet owners, veterinary clinicians, and the public community. Although the timeline for completion of a study in companion animals is longer than in rodents, completion remains rapid compared to human clinical trials as pets with aggressive and/or advanced stage cancer generally die within one year. Compliance is strong compared to human clinical trials, with repeat examination, treatment, and necropsy (autopsy) compliance approaching 90% [3]. 

## 2. Head and Neck Squamous Cell Carcinoma (HNSCC) Modeling

### 2.1. Feline Oral Squamous Cell Carcinoma (FOSCC): A Spontaneous Animal Tumor Model of HNSCC

Feline oral squamous cell carcinoma (FOSCC) has been suggested as a model for human head and neck squamous cell carcinoma (HNSCC), an aggressive cancer with limited advancement in five-year survival rates [7, 15, 16]. Feline models (tumor xenograft, normal cats, and cats with spontaneous disease) can be integrated dynamically with traditional preclinical models to optimize data value in drug testing. By utilizing pet cats that spontaneously develop oral squamous cell carcinoma (SCC), additional information could include drug activity and *in vivo* biomarkers, data in xenografts and clinical cancer patients, toxicity in cancer-bearing cats, PK/PD, drug dose and schedule, combination drug therapy, clinical monitoring, and tumor histology response. Other experimental animal models for human HNSCC include rodent 4NQO models, hamster tongue and check pouch models, and murine transgenic and xenograft models [17, 18]. These models all involve some form of artificial induction, underscoring the need for a relevant spontaneous model.

There have been limited translational and clinical advancements in HNSCC providing a rationale for alternative animal tumor models. Human head and neck cancer is the 8th most common cancer in the US and the 6th most common worldwide, with 40,250 new US cases/year [19]. As a histologic subtype HNSCC comprises approximately 90% of all head and neck cancers. Common initial management strategies include surgery and/or radiation in >65% of patients, with an increasing use of adjuvant chemotherapy [20]. Minimal improvement in overall survival has been made in the last two decades and HNSCC continues to have one of the worst outcomes of all head and neck tumors. For combined stages, the 1-year, 5-year, and 10-year relative survival rates are 84%, 61%, and 50%, respectively [19].

Feline oral squamous cell carcinoma (FOSCC) clinically mimics the disease progression and biologic behavior of HNSCC. Both cancers are locally invasive with metastasis occurring late in disease and most patients succumbing to local disease recurrence. In cats, reported metastases to regional lymph nodes (14.8%–18%) and lungs (12%) occur late in the course of disease and most cats are euthanized due to poor quality of life as a result of primary tumor growth [21, 22]. The incidence in cats is similar to people, with oral cancer accounting for up to 10% of all feline cancers [23]. Squamous cell carcinoma (SCC) is the most common oral tumor in cats (~60%–80%), occurring primarily in geriatric animals (12.5–13.6 years) [23, 24]. Gingiva is the most common intraoral site (51%), followed by lingual/sublingual (34%) [24]. Cats are the only species other than man that frequently develop SCC of the tongue [25]. Oral tumors in cats result in significant morbidity including chronic pain, malaligned dentition, halitosis, oral infection, dysphagia, food aversion, difficulty grooming, and cancer cachexia. 

### 2.2. Comparative Treatment Modalities: HNSCC and FOSCC

The most common initial management strategy for HNSCC includes surgery and/or radiation in >65% of patients [20]. Similar to people, local disease control is the mainstay of therapy in cats with FOSCC. Given the advanced stage at diagnosis in most feline patients, local surgical excision is not considered a curative treatment. The mandible is considered the anatomic region most amenable to surgical resection and when deemed feasible in select cases, mandibulectomy results in median survival times of 5–12 months [26–30]. Given the importance of both functional outcome and primary tumor control, surgical decisions must account for both morbidity and the likelihood of achieving complete excision. In one study, acute and long-term postoperative morbidity were observed in 98% and 76% of the cats, respectively, with 12% of cats never regaining functional ability to eat. Despite curative-intent surgical approaches, 48% of tumor specimens had evidence of residual disease histologically [30]. In one small study, the combination of mandibulectomy and radiation therapy (RT) resulted in a median survival of 14 months, suggesting that for select cases, a better long-term prognosis may be feasible [31].

Radiation therapy is the primary treatment modality for unresectable local disease. Radiation therapy as a single-agent therapy results in overall response rates of 54%–70%, median progression-free intervals of 1.8–3.5 m, and median survival times of 2–5.8 m [22, 32, 33]. Similar to human studies, in cats chemotherapy as a single agent for primary tumor control is considered ineffective [21]. However, adjuvant chemotherapy may have a role in both local tumor control as well as metastatic spread. Cisplatin is routinely used in HNSCC, and a similar protocol using a combination of radiation and carboplatin chemotherapy resulted in a median survival of 5.4 months in cats [34]. Given the hypoxic nature of these tumors, gemcitabine and etanidazole have been investigated in cats as hypoxia/radiosensitizers, with response rates of 75%–100% yet rapid progression with median survival times of approximately 3.7 months [35]. 

 Despite therapy, most cats are euthanized due to local disease progression and the severity of subsequent clinical signs. Overall, median survival times are generally between 2 and 4 months, with a one-year survival of only 10% [27, 29, 33, 36]. While disappointing, these numbers underscore the potential to institute investigational therapies in cats with rapid timelines for study completion.

### 2.3. Comparative Etiopathogenesis: HNSCC and FOSCC

The etiopathogenesis of HNSCC is multifactorial, with major risk factors including smoking/tobacco consumption, alcohol consumption, betel nut consumption (worldwide), poor oral hygiene, and human papillomavirus infection [37]. Recent studies suggest that human-papillomavirus- (HPV-) associated oropharyngeal cancers are increasing in incidence [19]. In people, oncogenic papillomaviruses alter retinoblastoma protein function, resulting in downstream accumulation of p16CDKN2A protein (p16). This subgroup accounts for ~20%–25% of oral cancers and is primarily associated with HPV-16. However, the clinical response and molecular properties of HPV-positive and HPV-negative tumors differ greatly, with HPV-negative HNSCCs having a worse overall survival [38]. In FOSCC, increased p16 immunoreactivity or papillomavirus DNA has been identified in only a small percentage of tissue samples, suggesting a potential role for PV-associated oncogenesis in a subset of FOSCC [39–41]. This suggests that FOSCCs are more similar to HPV-negative HNSCC, with no detectable papillomavirus DNA present in the majority of FOSCC [39–41]. Alternatively, it has been suggested by the “hit-and-run” mechanism of HPV-mediated carcinogenesis in HNSCC that perhaps HPV infection is an early initiating oncogenic event, but not necessary later for tumor progression, and thus HPV DNA may no longer be detectable [38]. However, papillomavirus DNA is detectable in more than 90% of feline cutaneous SCCs which carries a much better prognosis than FOSCC, similar to the clinical outcome for human cutaneous SCC and HNSCC [42]. Smoking and tobacco consumption are major risk factors for HNSCC. Second-hand tobacco smoke has also been implicated in the pathogenesis of FOSCC, with cats from smoking households at a two- to fourfold increased risk of oral squamous cell carcinoma compared with cats in nonsmoking households [43, 44]. Oral exposure to other chemicals via grooming is also thought to contribute to FOSCC pathogenesis, as cats chronically exposed to flea collars are at an increased risk compared to cats treated with intermittent flea shampoo [43]. In people, an increase in nasopharyngeal cancer has been associated with the consumption of salt-cured meats and fish [37]. Similarly, cats with high canned food intake (particularly tuna) are at increased risk of FOSCC [43]. The significance of this association is not clear and may be related to nutrient content (e.g., protein content) or a relationship between texture of food and oral hygiene. 

### 2.4. Comparative Molecular Aspects: HNSCC and FOSCC

#### 2.4.1. Epidermal Growth Factor Receptor

The epidermal growth factor receptor (EGFR) signaling pathway has been implicated in the oncogenesis of HNSCC. Epidermal growth factor receptor is a transmembrane receptor tyrosine kinase that controls cellular pathways crucial in cancer development, growth, invasion, metastasis, and angiogenesis. Up to 90% of HNSCC exhibit overexpression of EGFR, which is prognostic for overall survival and disease-free interval [37, 45]. EGFR-targeted therapies have been explored, with blocking of EGFR resulting in inhibition of cell proliferation, enhancement of apoptosis, and reduction in the metastatic and angiogenic potential of HNSCC. In addition, monoclonal antibodies (e.g., cetuximab) have been investigated in clinical HNSCC patients with modest results [37]. 

In cats with FOSCC, EGFR is similarly highly expressed (69%–100%) [46–48]. The significance of EGFR tumor expression is unclear, with two studies demonstrating either high or low EGFR scores associated with better survival [47, 48]. Limitations in comparison of these studies as well as comparison to human studies include the retrospective nature, small sample size, variability in patient treatment, and variability in method of EGFR scoring and cellular localization. *In vitro* in feline cells, EGFR has been associated with proliferation and migration [49]. A population of putative cancer stem cells with enhanced sphere-forming ability, reduced sensitivity to radiation and conventional chemotherapy, and demonstrated resistance to gefitinib (EGFR-targeting drug) has also been demonstrated in FOSCC [50]. Gefitinib results in reduced cell proliferation and migration as well as a change in cell morphology and gene expression suggestive of epithelial to mesenchymal transition [49, 50]. EGFR-targeting with RNAi resulted in reduction in EGFR activity, reversal of acquired gefitinib resistance, and an additive effect on cell killing when combined with radiation [49].

#### 2.4.2. Molecular Markers of Proliferation and Angiogenesis

HNSCC is characterized by tumor-associated angiogenesis, with elevations in the proangiogenic cytokine vascular endothelial growth factor (VEGF) and increased microvessel density (MVD) scores correlated with clinical stage and an overall poor prognosis [51]. In FOSCC, MVD is significantly higher in tumor samples (50.3 ± 23.6), compared with normal feline gingiva (7.6 ± 4.06) with tumor-associated vessels exhibiting morphologic atypia and a thin endothelial lining [52]. There may also be anatomic variability with tongue tumors reported to have the highest MVD [47]. In cats clinically affected with FOSCC, treatment with zoledronate, an aminobisphosphonate used for skeletal malignancies that has putative antiangiogenic properties, rapidly decreases circulating serum VEGF concentrations [16].

Prostaglandins and cyclooxygenase-2 (COX-2) play a critical role in tumor development and growth by regulating numerous biologic processes including tumor angiogenesis. More than 77% of HNSCC express high levels of COX-2 and COX-2 overexpression and higher prostaglandin E2 (PGE2) levels are associated with poor survival and correlated with VEGF expression [53, 54]. In FOSCC, COX-2 expression is variable, with up to 67% of tumors exhibiting COX-2 expression [55–57]. However, COX-2 expression is not reported to be correlated with survival [57]. Although the significance of this is unclear, functional COX-2 enzymatic activity is reported to be dissociated from relative IHC-based COX-2 protein expression in some cell lines including the FOSCC cell line SCCF1 [58]. In HNSCC, lipoxygenase is also overexpressed, and similarly in FOSCC tissue samples there is high-intensity staining of lipoxygenase. Additionally, *in vitro*, the 5-lipoxygenase inhibitor tepoxalin induces apoptosis in feline SCC cells [59]. 

The tumor suppressor gene p53 is often dysregulated in cancer, with loss of functional p53 protein contributing to disease progression via aberrant cell cycle checkpoint control and regulation of apoptosis. p53 gene mutation associated with allelic loss at 17p is one of the most common genetic abnormalities in HNSCC, and p53 status is an independent predictive factor of response to chemotherapy [60]. In HNSCC HPV-negative tumors, which have a worse overall survival, often have mutated p53, while the HPV-positive HNSCCs have both a better overall survival as well as wild-type p53 [38]. In addition, HPV-positive tumors tend to occur in younger people compared with HPV-negative HNSCC which commonly affects older patients. Similarly, most FOSCCs occur in geriatric cats and are HPV-negative [39, 40]. Aberrant p53 expression has been documented in FOSCC and is associated with tobacco smoke exposure [44, 61]. 

Ki67 and mitotic index (MI) are markers of proliferation commonly assessed in tumor biopsy samples. Ki67 is a nuclear protein not expressed in senescent cells (G0). In HNSCC, Ki67 expression is related to aberrant p53 expression and is associated with a poor prognosis [62, 63]. In FOSCC, high expression of Ki67 is associated with decreased overall survival [48]. In another study, both Ki 67 and mitotic index exhibited wide variation among tumors, with no correlation between MI and Ki 67 [47].

#### 2.4.3. Malignant Osteolysis

Both HNSCC and FOSCC are highly invasive into surrounding soft tissue structures and frequently characterized by malignant osteolysis of underlying bone. Bone-invasion contributes to clinical morbidity and poorer prognosis for HNSCC patients. In both species, *in vitro* and *in vivo* bone resorption and osteoclastogenesis are associated with high levels of parathyroid hormone-related protein (PTHrP) [15, 24]. PTHrP is known to stimulate osteoclastic bone resorption by increasing the expression of RANKL in osteoblasts. RANKL expression results in differentiation and activation of osteoclasts, ultimately resulting in bone resorption. SCC cells (murine, human, and feline) also express increased receptor activator of nuclear factor kappa-B ligand (RANKL) or an altered RANKL:OPG (osteoprotegerin) ratio [15, 52]. Osteoprotegerin is a decoy receptor for the receptor activator of nuclear factor kappa B ligand (RANKL); binding of RANKL by OPG inhibits nuclear kappa B (NF-*κ*B) downstream signaling. In feline SCC cells, the aminobisphosphonate zoledronate, clinically used in people to inhibit malignant osteolysis, induces a dose-dependent reduction in RANKL expression [16, 52]. In a murine xenograft (feline SCC cells), zoledronate treatment reduced tumor growth and prevented osteolysis [64]. Serum carboxy-terminal collagen crosslink (CTx) is a useful marker of bone resorption and turnover in clinical patients with skeletal malignancies. Cats with naturally occurring bone-invasive SCC have greater serum CTx concentrations in comparison with geriatric, healthy controls and in FOSCC-affected cats treatment with zoledronate rapidly decreased circulating serum CTx levels [16]. 

## 3. Conclusions

In conclusion, spontaneous animal tumor models may serve an important role in translating research findings to facilitate clinical improvements in human cancer patients. Spontaneously occurring cancer in pets shares striking molecular and clinical similarities to human malignancies that cannot be reproduced in artificially induced laboratory models. Human HNSCC is one type of cancer that has suffered from limited improvement in effective therapies and outcomes in the last few decades. Given the similarities in clinical progression and therapy as well as tumor biology including EGFR signaling, molecular aspects of cancer progression and angiogenesis, and clinical manifestations of malignant osteolysis, FOSCC is a biologically relevant animal tumor model. Utilization of this naturally occurring cancer may benefit both veterinary and human cancer patients. 
